# Soyo-san reduces depressive-like behavior and proinflammatory cytokines in ovariectomized female rats

**DOI:** 10.1186/1472-6882-14-34

**Published:** 2014-01-21

**Authors:** Hyun-Jung Park, Hyun-Soo Shim, Sun Yong Chung, Tae Hee Lee, Insop Shim

**Affiliations:** 1 Acupuncture and Meridian Science Research Center (AMSRC), Kyung Hee University,1 Hoegi-dong, Dongdaemun-gu, Seoul 130-701, Republic of Korea; 2 Department of Oriental Medical Science, College of Oriental Medicine, Graduate School, Kyung Hee University, Seoul 130-701, Korea; 3 Department of Oriental Neuropsychiatry, Kyung Hee University Korean Medicine Hospital at Gangdong, Seoul 130-701, Korea; 4 Department of Formulae Pharmacology, School of Korean Medicine, Graduate School of Gachon University, Seongnam, Gyeonggi-do 461-701, Korea

## Abstract

**Background:**

Soyo-san is a traditional oriental medicinal formula, a mixture of 9 crude drugs, and it has been clinically used for treating mild depressive disorders. The role of pro- and anti-inflammatory cytokines in psychiatric disorders has been the focus of great research attention in recent years. In the present study, we detected the antidepressant effect of soyo-san in the ovariectomized and repeated stressed female rats.

**Methods:**

This study was designed to evaluate the antidepressant-like effect of soyo-san on the forced swimming test (FST). The rats were randomly divided into the following groups: the nonoperated and nonstressed group (non-op), the nonoperated and stressed group (non-op + ST), the ovariectomized and stress group (OVX) and sham operated and stressed group (sham), the ovariectomized and stressed group (OVX + ST), the ovariectomized, stressed and soyo-san 100 mg/kg treated group (SOY100) and the ovariectomized, stressed and soyo-san 400 mg/kg treated group (SOY400). The rats were exposed to immobilization stress (IMO) for 14day (2 h/14day), and soyo-san (100 mg/kg and 400 mg/kg) was administrated during the same time. In the same animals, the levels of corticosterone and interleukin-1-beta (IL-1β) were examined in the serum. Also, the change of IL-1β expression in brain regions was examined after behavior test.

**Results:**

In the FST, the lower dose (100 mg/kg) of extract was effective in reducing immobility, along with an increase in swimming time. The serum levels of corticosterone and IL-1β in the SOY groups were significantly lower than those in the control group. In the brain, the expression of IL-1β positive neurons in the control group were significantly increased in the paraventricular nucleus (PVN) and hippocampus compared to the non-op. However, soyo-san groups significantly reduced the IL-1β-ir neurons in the PVN and hippocampal regions compared to the control.

**Conclusion:**

The present results demonstrated that soyo-san effectively reduced behavioral and patho-physiological depression-like responses. Trial registration: Our results suggest that soyo-san may be useful for immune regulator in repeated stress-induced ovariectomized female rats.

## Background

Postmenopausal women often suffer from symptoms called postmenopausal syndrome. These symptoms consist of hot flush and mental symptoms such as depression, irritation and insomnia [1,2]. Menopause is associated with a rapid decline in circulating sexual hormones and results in menopausal syndrome, including hot flushes, osteoporosis and affective disorders, for example, anxiety and depression. Those symptoms are known to be related to the decrease of the serum 17β-estradiol (E_2_) level [2,3]. The decrease of E_2_ has an influence on regulating the production of corticosterone and changing the behavioral response under the stress condition.

Repeated immobilization stress or unpredictable footshock elicit sickness behaviors, which may reflect part of a constellation of adaptive changes elicited by macrophage derived cytokines, such interleukin-1β and interferon-γ [4,5]. These cytokines may directly or indirectly affect CNS processes [6] and thus may have implications for psychopathology, including depressive illness if the increased level of pro-inflammatory cytokines is involved in the etiology of depression, it may be expected that antidepressants should have a restoring immunoregulatory effect. Pharmacological studies with adrenaline transporter (NET) inhibitors such as desipramine [7,8] have indicated the involvement of noradrenergic system underlying the effects of antidepressants on immune activity.

We already reported that the antidepressant effects of soyo-san on immobilization stress in ovariectomized female rats. The treatment with soyo-san caused significant reversals of the stress-induced deficits in learning and memory on a spatial memory task, and it also produced an anxiolytic-like effect on the EPM, and increased the ChAT and AchE reactivities. The serum level of corticosterone in the soyosan treated group was significantly lower than that in the control group. In this study, we aimed to investigate whether administration of soyo-san could modulate depressive–like behavior and production of pro-inflammatory cytokines. To achieve this goal, soyo-san’s antidepressant effect was tested via a forced swimming test (FST); moreover, the influence of soyo-san in producing of IL-1β was further assessed in the serum and brain regions using the ELISA and immunohistochemistry.

## Methods

### Subjects and stress procedure

Sprague Dawley female rats at the age of 3 months (Orient, Inc. Korea) were used for the study. The rats were housed under a controlled temperature (22-24°C) with a 12 h light/dark cycle. The lights were on from 8:00 to 20:00. Food and water were made available *ad libitum*. They were allowed at least 1 week to adapt to their environment before the experiments. The animal experiments were carried out in accordance with the Prevention of Cruelty to Animals Act 1986 and NIH guidance for the care and use of laboratory animals for experimental procedures, and all experiments in this study were approved by the Institutional Animal Care and Use Committee of Kyung Hee University (KHUAP(SE)-13-041).

The rats were randomly divided into the following groups: the nonoperated and nonstressed group (non-op), the nonoperated and stressed group (non-op + ST), the ovariectomized and stress group (OVX) and sham operated and stressed group (sham), the ovariectomized and stressed group (OVX + ST), the ovariectomized, stressed and soyo-san 100 mg/kg treated group (SOY100) and the ovariectomized, stressed and soyo-san 400 mg/kg treated group (SOY400). Using aseptic conditions, bilateral ovariectomy was performed under general anesthesia with pentobarbital sodium (50 mg/kg, i.p.). After postoperative recovery for 1 week, the ovariectomized rats were stressed daily. Stress was produced by forcing the animals into an immobilizer device (a disposable rodent restraint cone) for 2 hours (10:00–12:00 a.m.) for 2 weeks. The soyo-san group was orally administrated once daily for 2 weeks, and other groups were given sterile saline. Drug treatments began 30 min before the immobilization stress.

### Preparation of herbal extracts

Soyo-san was purchased from an oriental drug store (Jungdo Inc. Seoul, Korea). The boucher specimens are deposited at the herbarium located in the College of Oriental Medicine, Kyung Hee University. The dried soyo-san samples (720.58 g, Table 1) were immersed in a 10-fold volume of distilled water, boiled at 80°C for 1 h, and then the water extract was collected. The process was repeated once, and the extracts were combined and concentrated with a rotary evaporator and vacuum-dried to yield 6.6% (w/w) of the extract.

**Table 1:** Composition of soyo-san

**Medicinal plant**	**Amount (g)**
Paeoliae Radix Alba	4.0
Atractylodis Macrocephalae Phizoma	4.0
Angelicae Gigantis Radix	4.0
Poria	4.0
Liriopis Tuber	4.0
Bupleuri Radix	4.0
Menthae Herba	2.0
Glycyrrhizae Radix	2.0
Zingiberi Rhizoma Recens	6.0
Total	34.0

### Forced swimming test

From the 14^th^ day after the first immobilization, rats were placed in an FST, The time interval was 5 min. The FST was originally described by Porsolt et al. [9] and now is the most widely used pharmacological model for assessing antidepressant activity [10]. The development of immobility when the rodents are placed in an inescapable cylinder of water reflects the cessation of persistent escape-directed behavior [11]. The apparatus consisted of a transparent Plexiglas cylinder (50 cm high × 20 cm wide) filled to a 30 cm depth with water at room temperature. In the pre-test, rats were placed in the cylinder for 15 min, 24 h prior to the 5-min swimming test. Soyo-san extract (100, 400 mg/kg) or saline was administrated p.o. three times: immediately after the initial 15 min pre-test, 5 and 1 h prior to the swimming test. During the 5-min swimming test, the following behavioral responses were recorded by a trained observer: Climbing behavior, which is defined as upward-directed movements of the forepaws along the side of the swim chamber. Swimming behavior, defined as movement throughout the swim chamber, which included crossing into another quadrant. Immobility was considered when the rat made no further attempts to escape except the movement necessary to keep its head above the water. Increases in active responses, such as climbing or swimming, and reduction in immobility, are considered as behavioral profiles consistent with an antidepressant-like action [10].

### Corticosterone and cytokine measurements

After the behavior test, blood sample was taken in heart and then plasma separated from the blood was used to estimate the cytokine levels. Enzyme-linked immunosorbent assay (ELISA) was performed using Quantikine ELISA development system according to the manufacturer’s instructions (R & D Systems, Minneapolis, MN, USA). Briefly, polystyrene microtiter plates (NUNC, U16 Maxisorp type, Roskilde, Denmark) were coated with monoclonal antibody (anti-rat IL-1β and corticosterone) obtained from rat (R & D Systems) and incubated at 4°C overnight. The following day, the plates were blocked and then incubated for 2 h with plasma. This was followed by the addition of corresponding biotinylated detection antibody obtained from rat (R & D Systems) and incubated for 2 h. Streptavidin horseradish peroxidase (R & D Systems) and, then, tetramethylbenzidine substrate (Bangalore Genei, Bangalore, India) treatment followed this incubation. The reaction was stopped using 2 N sulfuric acid, and optical density reading was taken at 450 nm (ELISA reader: Organon Teknika Microwell system, Reader 230 s, Eppelheim, Germany). All the experiments were conducted in duplicate. A standard curve was obtained based on the standards provided by the manufacturer. The results were expressed as concentration of cytokines (in pg/ml) read from the standard curve.

### IL-1β immunohistochemistry

After the behavioral testing was completed, all of the animals were deeply anesthetized with sodium pentobarbital (80 mg/kg, i.p.) also, blood sample was taken in heart and then they were perfused through the ascending aorta with normal saline (0.9%), followed by 800 ml of 4% paraformaldehyde in 0.1 M phosphate buffer saline (PBS). The brains were removed, postfixed overnight and cryoprotected in 20% sucrose in 0.1 M PBS at 4°C. The brains were cut by cryostat sectioning into 30 μm coronal sections at the level of the hippocampus, and these slices were processed histochemically as free-floating sections.

The brain sections were washed in PBS containing 0.3% Triton X-100. The primary goat polyclonal antibodies against the following specific antigen were used: Intetleukin-1-beta (IL-1β, concentration 1:100; R&D Systems, Minneapolis, USA). The primary antibodies were diluted with blocking solution (10% fetal bovine serum in PBS, pH = 7.4) and the tissues were incubated for 72 h at 4°C with constant agitation. Following rinsing in PBS, the sections were incubated for 2 h at room temperature in biotinylated goat anti-serum (Vector Laboratories, Burlingame, CA) that was diluted 100:1 in PBST containing 2% normal rabbit serum. The sections were placed in Vectastain Elite ABC reagent (Vector Laboratories, Burlingame, CA) for 2 h at room temperature. Following a further rinsing in PBS, the tissue was developed using diaminobenzidine chromogen with nickel intensification. The sections were mounted on gelatine-coated slides, air-dried and coverslipped for microscopic observation. For measuring the cells, a microrectangular grid (200 × 200um) was placed according to the atlas of Paxinos and Watson under the light microscope (×100 magnification) [12].

### Data analysis

Statistical comparisons were done for the behavioral, histochemical and immunological studies using the one-way ANOVA, respectively, and Tukey *post hoc* was done. All of the results were presented as means ± S.E.M., and we used SPSS 15.0 for Windows for analysis of the statistics. The significance level was set at *p* < 0.05.

## Results

### Forced swimming test

**Figure 1:**
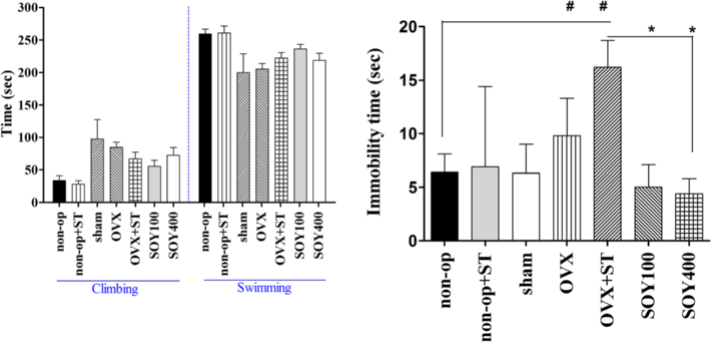
**The effects of soyo-san extract on CORT (A), IL-1β (B) levels in the serum.** The results of ELISA were analyzed by performing separate one-way ANOVA among the groups. Each value represents the mean ± S.E.M. *P < 0.05, **P < 0.01 compared to OVX +ST. #*P* < 0.05, ##*P* < 0.01 compared to non-op.

The effect of the soyo-san on active behaviors in the FST of rats are shown in Figure 1. ANOVA revealed effects of the treatment of soyo-san on immobility, F_3,30_ = 5.214, *P* < 0.05; swimming behavior, F_3,30_ = 0.328; and climbing behavior, F_3,30_ = 0.150. Post hoc analysis demonstrated that soyo-san treatment groups significantly shortened the immobility time in comparison to control values (*P* < 0.05). This effect was accompanied by increases in swimming behavior, after 100 and 400 mg/kg. These results indicated that higher doses (400 mg/kg) were more effective than soyo-san 100 mg in reducing immobility time.

### ELISA

1) Corticosterone (CORT)

**Figure 2:**
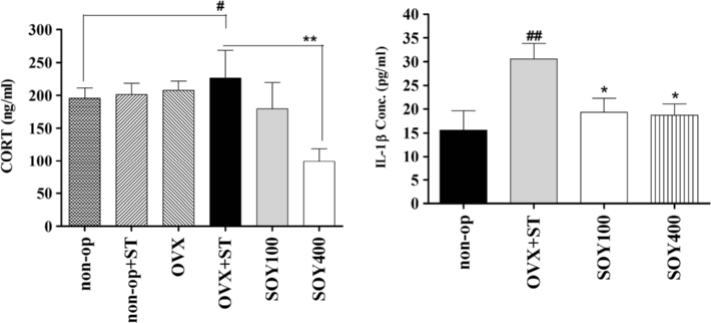
**The climbing and swimming time (A) and the immobility time (B) of rats on the forced swimming.** Data represent means ± S.E.M. of the duration of climbing, swimming and immobility during the 5-min test session. The results of FST were analyzed by performing separate one-way ANOVA of mean counts among the groups. Each value represents the mean ± S.E.M. *P < 0.05 compared to OVX+ST. *#P < 0.05* compared to non-op.

Figure 2A shows that the serum levels of CORT were significantly different in comparisons among the groups (F_3,30_ =4.093, *P* < 0.05). The serum levels of CORT were 195.6 ± 15.4, 226.3 ± 42.4, 179.1 ± 40.3 and 99.1 ± 19.6 (ng/ml) in the non-op, OVX+ST, SOY100 and SOY400 groups, respectively. The LSD test results indicated a significantly increased the serum levels of CORT in the non-op group compared to the OVX+ST group (*P* < 0.05). After treatment of soyo-san, the serum levels of CORT were significantly decreased in the SOY100 group (*P* <0.05) and SOY400 group (*P* < 0.01) compared to the OVX+ST group.

2) IL-1β

Figure 2B. shows that the serum levels of IL-1β were significantly different in comparisons among the groups (F_3,30_ =4.1, *P* < 0.05). The serum levels of IL-1β were 15.6 ± 4.0, 30.6 ± 3.3, 19.3 ± 3.0 and 18.7 ± 2.4 (pg/ml) in the non-op, OVX+ST, SOY100 and SOY400 groups, respectively. The LSD test results indicated a significantly increased the serum levels of IL-1β in the non-op group compared to the stressed group (*P* < 0.01). After treatment of soyo-san, the serum levels of IL-1β were significantly decreased to 33.0% of the stress in the soyo-san groups (*P* < 0.05).

### IL-1β Immunohitochemistry

**Figure 3:**
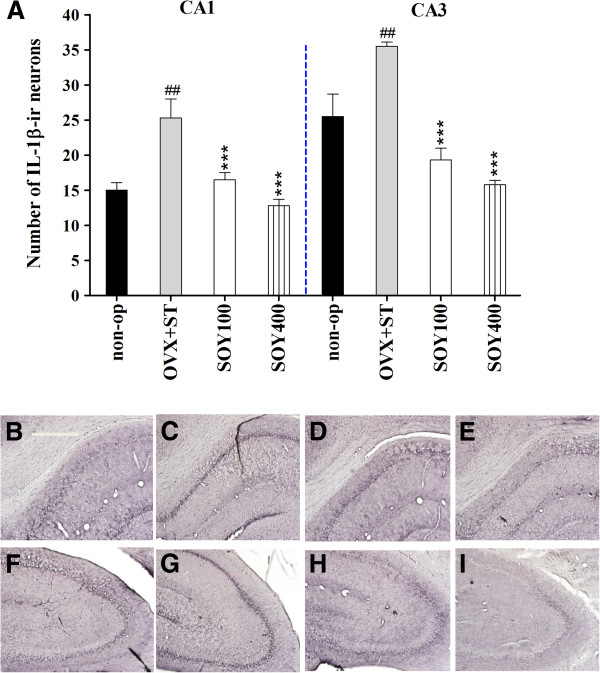
**The effect of soyo-san extract on quantities of IL-1β immunostained nuclei expression in the hippocampal regions of the experimental groups after forced swimming test. (A)** The results of IL-1β-reactivity were analyzed by performing separate one-way ANOVA of neurons among the groups. Each value represents the mean ± S.E.M. ***P < 0.001 compared to OVX+ST. ##*P* < 0.01 compared to non-op. **B-I**: Representative photographs of IL-1β-immunostained brain sections. Scale bars, 200 um. non-op **(B,F)**, OVX+ST **(C,G)**, SOY 100 **(D,H)** and SOY 400 **(E, I)**.

**Figure 4:**
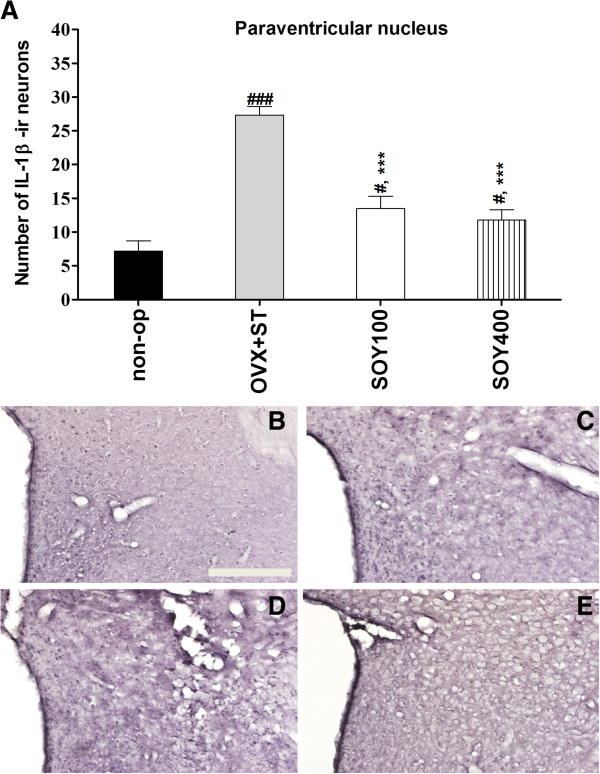
**The effect of soyo-san extract on quantities of IL-1β immunostained nuclei expression in the paraventricular regions of the experimental groups after forced swimming test. (A)** The results of IL-1β-reactivity were analyzed by performing separate one-way ANOVA of neurons among the groups. Each value represents the mean ± S.E.M. ***P < 0.001 compared to non-op. #*P* < 0.05, ###*P* < 0.001 compared to non-op. **B-E** Representative photographs of IL-1β-immunostained brain sections. Scale bars, 200um. non-op **(B)**, OVX +ST, SOY 100 **(D)** and SOY 400 **(E)**.

The results of determining the IL-1β immunoreative cells per section from different hippocampal formations and paraventricular nucleus are shown in Figures 3 and 4. The number of IL-1β neurons in the CA1 area was 10.1 ± 0.6 in the non-op group, 3.9 ± 0.6 in the OVX group, 3.0 ± 0.3 in the SOY100 group and 6.0 ± 0.4 in the SOY400 group [F_3,20_ = 11.7, *P* < 0.001; 3A-I]. IL-1β immunoreactive cells in the CA3 area were 18.4 ± 0.6 in the non-op group, 13.7 ± 0.8 in the OVX group, 12.9 ± 0.3 in the SOY100 group and 15.7 ± 0.5 in the SOY400 group [F_3,20_ = 6.0, *P* < 0.01].

Also, the number of IL-1β positive neurons was significantly increased to 121.7% of the OVX in the non-op group (*P* < 0.01). The number of IL-1β neurons in the PVN area was 7.2 ± 1.5 in the non-op group, 27.3 ± 1.3 in the OVX group, 13.5 ± 1.8 in the SOY100 group and 11.8 ± 1.5 in the SOY400 group [F_3,20_ = 32.4, *P* < 0.001; 4A-E].

## Discussion

The present study demonstrated that the administration of soyo-san reduced the depressive behavior and pro-inflammatory cytokine expression. These results suggest that repeated immobilization stress in the ovariectomized female rats is an immune challenge capable of inducing depressive-like behavior, promoting exaggerated glucocorticoid responses to stress, and changing cytokine transcription in the serum and the brain.

Depression is defined clinically as a pathological complex of psychological, neuroendocrine and somatic symptoms that cannot be reproduced in animals. To date, few models are commonly used for screening antidepressant effects or studying the mechanisms of action of these molecules. The forced swimming test (FST) was discussed the main parameters that influence the sensitivity, specificity and reliability of this model. Porsolt et al. (1977) [13] described “a new behavioural method for inducing a depressed state”. The idea arose out of some learning experiments they were doing with rats in a water maze. Most rats were finding the exit within 10 min but they noticed that other rats ceased struggling altogether and remained floating passively [9]. The duration of immobility occurring in each minute was scored. A wide variety of antidepressants, and compounds with potential antidepressant activity, reduce the duration of immobility in the forced swimming test [14-16]. The present study confirms the previously reported antidepressant like effect of soyo-san. The reduction in immobility induced by soyo-san was accompanied by increased swimming, while that of soyo-san occurred with an increase in climbing behavior. The behavioral profiles observed in the present study with soyo-san, in ovariectomized female rats, are similar to those reported by other authors in male rats [17-21]. Hence, the finding of Chen et al. [22] can also be extended to repeated stress induced depression animal model. In a Previous study, we also demonstrated that Bupleuri radix, one of the most important constituents of soyo saan, caused a significant anti-immobility effect on the FST and reversed the CMS-induced reduction of sucrose consumption [23]. In agreement with these findings, the previous studies on major depressive disorder have reported the elevation of IL-1β production during a depressive state [24]. This study also showed the repeated immobilization stress induced a broad spectrum of behavioral changes that might be regarded as depression-like symptoms, including reduction of preferences for sucrose solution, inhibition of locomotor activities, and induction of learned helplessness behavior.

Several lines of evidence have indicated that menopause is associated with increased susceptibility to neurological disorders [25], also the mechanisms were elucidated [25-27]. Previous studies revealed a substantial involvement of endogenous estrogen in neuroinflammatory processes and provide novel mechanisms for hormone action in the brain [25-27]. Another study reported that the estrogen replacement therapy (ERT) was related to exacerbate the memory impairment induced by the chronic neuroinflammation associated with Alzheimer’s disease [27]. In this study, our aim is to investigate whether OVX animals are more vulnerable to the inflammatory and behavioral effects of chronic stress. Many studies proved that the repeated stress accelerated expression of pro-inflammatory cytokines in the brain [28-30]. Pro-inflammatory cytokines play a key role in determining the nature and strength of immune responses [31]. Previous studies have demonstrated that the induction of pro-inflammatory cytokines activated under stress condition. Stress (physical, psychological or both of them) can increase an inflammatory responses. The relationship between stressful events and the onset of inflammatory related diseases (injury, ischemia, Alzheimer’s disease) is well documented [32]. Stress response is very similar to the inflammatory process generated in an organism when invaded by certain microorganisms or after injury or tissue damage as oxidative stress. In rodents, unavoidable physical stresses are the most extensively used: immobilization, exposure to extreme temperatures, fasting, immersion or electric foot shock. Various studies have provided inputs on the molecular signaling that links stress-induced neuro-endocrine-immune interactions, suggesting a bi-directional crosstalk which is based on the secretion of cytokines, glucocorticoids, neurotransmitters and neuropeptides [33]. The previous study proved that the sex difference in novelty exposure observed in the form of a greater hypothalamic-pituitary-adrenal (HPA) axis response in female ICR mice is controlled by ovary-derived factors in adults [34]. Ovariectomy expresses high levels of adrenal steroid receptors and is susceptible especially to damage as a result of stress [35]. Women have a higher incidence of some stress-related disorders, such as depression, while men are generally at a greater risk for cardiovascular diseases, such as myocardial infarction and hypertension, than age-matched, premenopausal women [36]. The higher incidence and severity of depression are associated with the presence or absence of ovarian hormones [37]. The present study showed hyperactivity of the pro-inflammatory cytokine after stress. Pro-inflammatory cytokine’s overproduction could be integrated in the inflammatory response system, which is activated during depression, and this is consistent with the shift of the pro-/anti-inflammatory cytokine mechanism. Buchanan et al. reported that psychological stress increases the brain levels of inflammatory cytokines such as interleukin-1β (IL-1β), IL-6 and tumor necrosis factor α (TNF-α) [38]. Zili et al. detected depression like behavior in a rat animal model which was induced inflammation in the spleen and brain by chronic mild stress (CMS) [39]. In the present study, we observed that the expression of IL-1β immunoreactive cells was up-regulated in paraventricular nucleus (PVN), motor trigeminal nucleus (MTN), hippocampus after repeated immobilization stress. The results from depression-like animal model were consistent with previous reports, which indicated that life events and depressive symptoms are associated with the rise of central cytokine such as IL-1β in human MDD patents [40-47] and stress-treated animals [48-50]. However, the administration of soyo-san significantly reduced the expression of IL-1β. *Menthae herbam*, a component of soyo-san modified prescription on plasma metabolomics of rats with chronic immobilization stress [51]. *Angelicae gigantis radix*, a component of soyo-san, exhibited an anti-inflammatory effect in vitro, in vivo and inhibits the stress-induced pathophysiological changes in the central nervous system [52]. Also, paeoniflorin has neuroprotective effect and inhibition of neuroinflammation [53].

## Conclusion

In conclusion, soyo-san decreased the immobility time in FST. Soyo-san also inhibited repeated stress induced IL-1β production in hippocampus and PVN. These results have shown that IL-1β, a pro-inflammatory cytokine, is an important molecule in the modulation of depressive-like behavior. This finding calls for a reappraisal of the “cytokine hypothesis of depression”. Further studies are encouraged to analyze the therapeutic effect of different levels of estrogenic compounds, which lack feminine proprieties, on different setting of immune diseases.

## Competing interests

The author declares that they have no competing interests.

## Authors’ contributions

HS and HJ conducted the animal experiment and analyzed the data. IS, HJ, SY and TH participated in design of the study and preparation of the manuscript. All the authors read and approved the final manuscript.

## Pre-publication history

The pre-publication history for this paper can be accessed here:

http://www.biomedcentral.com/1472-6882/14/34/prepub
